# Recent Advances and Challenges of mTOR Inhibitors Use in the Treatment of Patients with Tuberous Sclerosis Complex

**DOI:** 10.1155/2017/9820181

**Published:** 2017-03-12

**Authors:** Filipe Palavra, Conceição Robalo, Flávio Reis

**Affiliations:** ^1^Centre for Child Development, Neuropediatrics Unit, Pediatric Hospital, Coimbra Hospital and University Centre, Coimbra, Portugal; ^2^Laboratory of Pharmacology & Experimental Therapeutics, Institute for Biomedical Imaging and Life Sciences (IBILI), Faculty of Medicine, University of Coimbra, Coimbra, Portugal; ^3^Centre for Neuroscience and Cell Biology-Institute for Biomedical Imaging and Life Sciences (CNC.IBILI) Research Consortium, University of Coimbra, Coimbra, Portugal

## Abstract

Tuberous sclerosis complex (TSC) is a genetic condition characterized by the presence of benign, noninvasive, and tumor-like lesions called hamartomas that can affect multiple organ systems and are responsible for the clinical features of the disease. In the majority of cases, TSC results from mutations in the* TSC1* and* TSC2* genes, leading to the overactivation of the mammalian target of rapamycin (mTOR) signalling pathway, which controls several cell functions, including cell growth, proliferation, and survival. The establishment of a connection between TSC and mTOR led to the clinical use of drugs known as mTOR inhibitors (like rapamycin, also known as sirolimus and everolimus), which are becoming an increasingly interesting tool in the management of TSC-associated features, such as subependymal giant cell astrocytomas, renal angiomyolipomas, and also epilepsy. However, the intrinsic characteristics of these drugs and their systemic effects in such a heterogeneous condition pose many challenges in clinical practice, so that some questions remain unanswered. This article provides an overview of the pharmacological aspects of mTOR inhibitors about the clinical trials leading to their approval in TSC-related conditions and exposes current challenges and future directions associated with this promising therapeutic line.

## 1. Introduction

Tuberous sclerosis complex (TSC) is an autosomal dominant genetic disorder of cellular differentiation and proliferation, which is characterized, in pathological terms, by the presence of benign and noninvasive tumor-like lesions (called hamartomas) that can affect multiple organ systems, such as the brain, kidney, skin, heart, lung, and liver [1]. Hamartomas are then responsible for many of the clinical features of TSC, but true neoplasms also occur, particularly affecting the kidney and the brain.

Population-based studies suggest that TSC affects both children and adults, with an estimated incidence at birth of approximately 1 in 6000 [2, 3] and a prevalence between 1 : 14.000 and 1 : 25.000 [4, 5]. However, because of the striking variability and severity of clinical presentation, the diagnosis can be difficult to establish in individuals with subtle findings and the true prevalence may be higher. Patients are most frequently diagnosed with less than 15 months of age and evidence points that TSC prevalence decreases as age increases, being of 1 : 14.000 for those aged less than 6 years, 1 : 19.000 at 12 years, and 1 : 24.000 at 18 years old [4, 5]. Cardiac and cutaneous findings are usually the first clue that a patient has TSC, but many other features may lead to the diagnosis, which is currently based upon clinical characteristics and/or genetic testing, as coming from the International Tuberous Sclerosis Complex Consensus Conference, held in 2012 [6]. The following summarizes the clinical diagnostic criteria for TSC, including 11 major and 6 minor features (adapted from [6], where *∗* denotes that a combination of lymphangioleiomyomatosis and angiomyolipomas with no other clinical features does not meet criteria for a definite diagnosis (it is considered as only 1 major feature)).

*Major Clinical Features*

- Hypomelanotic macules (≥3, at least 5 mm diameter)
- Angiofibromas (≥3) or fibrous cephalic plaque
- Ungual fibromas (≥2)
- Shagreen patch
- Retinal hamartomas (multiple)
- Cortical dysplasia (≥3, including tubers and brain white matter radial migration lines)
- Subependymal nodules
- Subependymal giant cell astrocytoma
- Cardiac rhabdomyoma
- Lymphangioleiomyomatosis^*∗*^
- Angiomyolipomas (≥2)^*∗*^

*Minor Clinical Features*

- “Confetti” lesions of the skin (hypomelanotic macules with 1-2 mm)
- Dental enamel pits (≥3)
- Intraoral fibromas (≥2)
- Retinal achromic patch
- Renal cysts (multiple)
- Nonrenal hamartomas

For a definite diagnosis to be made, in clinical grounds, two major features are required or, alternatively, one major and two or more minor features. Possible TSC can be considered when one major feature or two or more minor features are present. However, genetic testing alone (the identification of either a* TSC1* or* TSC2* pathogenic mutation in DNA extracted from nonlesional tissue) is sufficient to make a definite diagnosis of TSC.

In fact, in this condition, mutations in one of the two tumor suppressor genes,* TSC1* (9q34, encoding hamartin) or* TSC2* (16p13.3, adjacent to the gene of adult polycystic kidney disease and encoding tuberin), are found in more than 85% of the cases [7]. These two proteins (hamartin and tuberin) form a single functional unit that is involved in the regulation of cell proliferation and differentiation—their complex activates GTPase, keeping the RHEB (Ras homolog enriched in brain) protein inactive, inhibiting the mammalian target of rapamycin (mTOR) pathway [1, 7]. This pathway promotes protein and lipid biosynthesis and is also responsible for cell cycle progression, playing a crucial role in cell proliferation [8]. Therefore, in TSC patients,* TSC1* or* TSC2* mutations give rise to hyperactivation of the mTOR pathway, inducing several abnormalities in numerous cell biochemical processes, including cell cycle regulation and control at transcriptional, translational, and metabolic levels.

Given this underlying abnormality in TSC, the possibility of using the mTOR pathway as a therapeutic target has been investigated, namely, using mTOR inhibitors, such as sirolimus (or rapamycin) and everolimus, firstly as an alternative nonsurgical intervention for subependymal giant cell astrocytomas (SEGA) in TSC patients. In fact, resulting from this research, everolimus is currently the only mTOR inhibitor approved in various countries for the treatment of patients with more than 3 years of age with TSC-related SEGA who are not candidates for curative surgical resection [9] and adults with TSC-associated renal angiomyolipomas who are at risk of complications, but who do not require immediate surgery [10].

This pharmacological strategy opened research avenues in the field of TSC and, in recent years, many scientific achievements have been obtained, for patients benefit. However, many challenges came along and, still, many disease features are poorly understood. This review will focus on the role of mTOR inhibitors in the treatment of TSC patients. After discussing the relevance of mTOR pathway in the disease, some pharmacological issues of mTOR inhibitors will be focused, from trials to clinical practice. Future perspectives and challenges will be also addressed.

## 2. Overview of mTOR-TSC Regulation

Mammalian target of rapamycin (mTOR) is an evolutionarily highly conserved serine/threonine protein kinase, member of the phosphoinositide 3-kinase- (PI3K-) related kinase family and of cell survival pathways. mTOR integrates extracellular and intracellular events to act as a molecular sensor of nutrient abundance and energy, thus having a major impact on a variety of functions in distinct organs and related clinical disorders [11–13]. Under normal conditions or disease states, mTOR activation by phosphorylation, in response to various upstream modulators of mTOR kinase (such as nutrients, growth factors, hormones, and mitogens), contributes to regulating several key processes to cell functioning [8].

**Figure 1:**
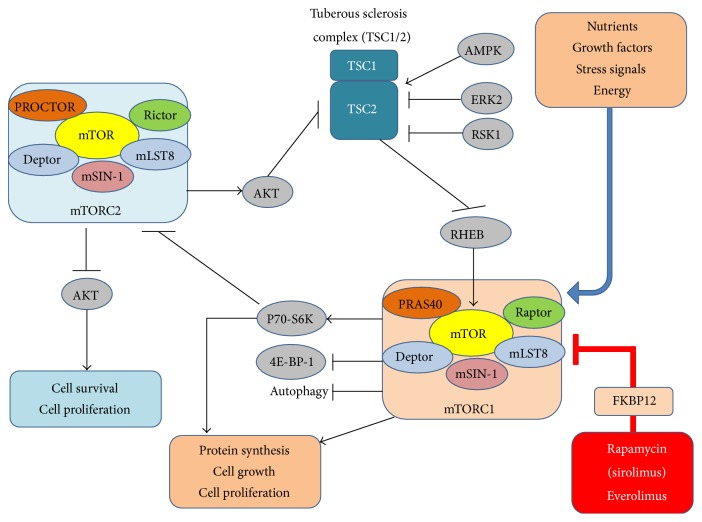
Overview of mTOR-TSC regulation and upstream and downstream mediators. Both TSC1 and TSC2 are major components in mTOR signalling cascade. mTOR complexes 1 and 2 (mTORC1 and mTORC2, resp.) are mediators of important cellular functions: mTORC1 (which senses nutrients, energy, growth factors, and stress signals) promotes protein synthesis, cell growth, and cell proliferation, while mTORC2 is associated with cell survival and proliferation. Tuberous sclerosis complex patients present mutations in either* TSC1* or* TSC2* genes, causing suppression of RHEB-mediated mTORC1 inhibition, exacerbating cell cycle progression, cell proliferation, and growth. Rapamycin (sirolimus) and everolimus are effective inhibitors of mTORC1 via FKBP12.

Two complexes (mTORC1 and mTORC2), with different composition, control mTOR actions [8, 14, 15] (Figure 1). There are two common proteins shared by both complexes: mLST8 (mammalian lethal with Sec13 protein 8, also known as G*β*L), which is a positive regulator, and DEPTOR (DEP-domain containing mTOR-interacting protein), which works as the negative regulator. Regarding specific components, mTORC1 is associated with Raptor (regulatory-associated protein of mTOR), a positive regulator involved in substrate recruitment, and PRAS40 (proline-rich AKT substrate of 40 kDa), the component responsible for mTORC1 inhibition, which is itself inhibited by Akt; mTORC2 is associated with Rictor (rapamycin-insensitive companion of mTOR), an essential player in the activation of the interaction between mTORC2 and tuberous sclerosis complex 2 (TSC2), with mSIN-1 (mammalian stress-activated protein kinase interacting protein), which is necessary for the assembly of the complex and for its capacity to phosphorylate Akt, and with PROTOR-1 (protein observed with RICTOR-1), which has been shown to bind to RICTOR and seems to play a role in enabling mTORC2 to efficiently activate serum- and glucocorticoid-induced kinase 1 (SGK1) [14–17] (Figure 1).

mTORC1 and mTORC2 complexes also differ in sensitivity to rapamycin, which binds to protein FKBP12 (FK 506-binding protein of 12 kDa), thus inhibiting mTOR phosphorylation and activation, preferentially at the mTORC1 complex [18]. Furthermore, mTORC1 and mTORC2 are distinctly regulated and control different cell processes: while mTORC1 senses growth factors, mitogens, nutrients (amino acids and energy), and stress signals, thus regulating cell growth and proliferation, mTORC2 is linked with cell survival and cycle progression, being insensitive to nutrients or cellular energy [15, 18]. Tumor suppressor proteins hamartin and tuberin form a heterodimeric complex (TSC1/2) that acts as a functional unit in the suppression of mTORC1 activity [7] (Figure 1). Nutrients are positive regulators by putatively acting upstream of TSC1/2; in addition, mTORC1 senses cellular energy status via the AMP-activated protein kinase (AMPK) and TSC2 [19]. In fact, while, under conditions of low energy, AMPK activation promotes mTOR inhibition via TSC2 phosphorylation, under hypoxic conditions, mTORC1 is inhibited by TSC1/2 and/or AMPK-dependent mechanisms [20, 21]. Although the mTORC2 upstream modulators remain to be fully elucidated, activation has been linked with phosphorylation of Akt and other AGC-family kinases, including serum- and glucocorticoid-induced protein kinase (SGK) and protein kinases C (PKCs), which play a central role in cell survival and cytoskeleton organization [8]. Akt appears to have a complex dual role on mTOR, being both an upstream regulator of mTORC1 and a downstream target of mTORC2. mTOR-dependent phosphorylated Akt is responsible for the regulation of several cellular processes, including cell growth, proliferation, apoptosis, and glucose metabolism [8]. Some of the downstream targets of mTORC1 were already clearly identified, including S6Ks (p70 ribosomal protein S6 kinase 1/2) and 4E-BPs (eukaryotic initiation factor 4 [eIF4] binding proteins), which play a major role in the regulation of cell growth, proliferation, and metabolism [15] (Figure 1). In addition, mTOR has also been involved in the regulation of other proteins which play major physiological and pathophysiological (namely, in tumorigenesis) roles, such as the hypoxia-inducible factor 1*α* (HIF-1*α*), which is a major player in angiogenesis, inflammation, bioenergetics, proliferation, and apoptosis, and the STAT3, which is involved in the effects mediated by several cytokines (such as IL-6 and IL-10) [18, 20–24].

A decrease in mTOR signalling in response to cellular stresses, such as low ATP or oxygen levels, as well as nutrient or amino acid depletion is an important autophagy initiator. This works as a conservative action to mitigate cellular injury. The inhibition of mTOR using rapamycin induces early activation of the autophagy cascade and this can also be a mechanism of interest in the treatment of several degenerative diseases, in which defects in autophagic clearance of cellular proteins have been implicated, and also in the oncology field, since this seems to be strongly implicated in tumorigenesis [25]. mTORC1 is an inhibitor of autophagy, via ULK1 and ULK2 kinases and, by using the mTORC1 inhibitor rapamycin and the autophagy inhibitor chloroquine in vitro, Yu et al. [25] demonstrated that TSC2-deficient cells are highly dependent on autophagy for survival. In fact, TSC provides a good model into the roles of autophagy in human disease but also indicates the possibility of using autophagy inhibition as a therapeutic target, namely, in combination with mTORC1 inhibitors, as was also suggested for lymphangioleiomyomatosis (LAM) [26], a condition also associated with mTOR activation and TSC2 gene mutations. In agreement, Parkhitko et al. [27] showed that the combination of mTORC1 and autophagy inhibition was more effective, when compared with either treatment alone, in inhibiting the survival of tuberin- (TSC2-) null cells, growth of TSC2-null xenograft tumors, and development of spontaneous renal tumors in Tsc2(+/−) mice. These authors suggested that mTORC1 inhibitors may have autophagy-dependent prosurvival effects in TSC and that autophagy and the autophagy target p62/sequestosome 1 could be viewed as two distinct therapeutic targets for TSC [27]. More recently, combined strategies of mTOR inhibition (with rapamycin) and autophagy inhibition (with resveratrol, a naturally occurring polyphenol) showed a selective induction of apoptosis in TSC2-deficient cells [28]. Using that strategy, the rapamycin-induced upregulation of autophagy was blocked and Akt inhibition restored. It was concluded that the combination of rapamycin and resveratrol could be viewed as an effective therapeutic approach for treatment of diseases with mTORC1 hyperactivation, such as TSC and LAM [28, 29]. This strategy could be very important to overcome one of the major concerns with the use of mTORC1 inhibitors, the upregulation of autophagy and the suppression of the negative feedback loop to Akt, which stimulates cell survival, thus reducing the efficacy of therapy and increasing the possibility of relapses upon cessation of treatment [28, 29].

## 3. Rapamycin and Analogues

**Figure 2:**
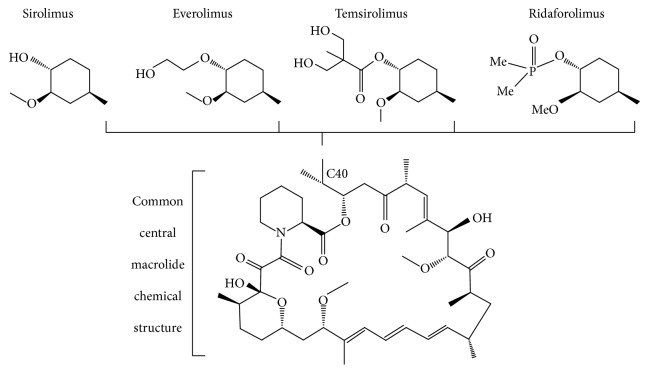
Molecular structure of sirolimus and its analogues. They all share a central macrolide chemical structure and have a unique R group at the C40 position.

Rapamycin, also known as sirolimus, is a natural macrolide firstly isolated from a bacteria strain of the* Streptomyces* genus* (Streptomyces hygroscopicus)* from soil bacterium extracts found on Easter Island. Rapamycin (or Rapa Nui, the native name) was initially described as an antibiotic and antifungal agent, but its potent immunosuppressant properties meanwhile discovered led to its approval (in 1999) as a drug for preventing allograft rejection [30]. In addition, rapamycin has demonstrated several other interesting effects, including cytostatic and antiproliferative properties, expanding the potential clinical applications to oncology. In fact, rapamycin and its derivatives meanwhile developed, collectively referred to as rapamycin analogues (everolimus, temsirolimus, and ridaforolimus), have been used or tested in several types of cancers, such as in advanced renal cell carcinoma, bladder, breast, neuroendocrine tumors, and inclusively TSC-related SEGA and renal angiomyolipomas [31–33].  Figure 2 represents the molecular structure of rapamycin (sirolimus) and its analogues.

### 3.1. Pharmacodynamic Properties and Clinical Applications

The mechanisms of action for sirolimus and analogues are similar and involve the formation of a complex by interacting with the intracellular binding protein FK506-binding protein (FKBP12), which then binds to mTOR at the FKBP12-rapamycin binding domain, thus inhibiting downstream signalling events (Figure 1). Sirolimus mechanism of action was first revealed using* Saccharomyces cerevisiae*; the binding to FKBP, involving both* TOR1* and* TOR2* genes, was responsible for arresting yeast in the G1 phase of the cell cycle [34]. In mammalian cells, the complex is relatively different since TOR (mTOR) exists as a single 289 kDa isoform that specifically binds to FKBP12. Rapamycin (sirolimus) does not directly bind to mTOR, but it is the highly selective binding of rapamycin to FKBP12 that mediates FKBP12 dimerization with mTOR, thus blocking the access to the mTOR kinase active site, causing a highly sensitive mTORC1 inhibition [34]. This feature is common to sirolimus and its analogues; shared macrolide structure allows the interaction with FKBP12, the mechanism by which these allosteric molecules selectively inhibit mTORC1 over mTORC2. Both sirolimus and its analogues exert their inhibitory effects on mTOR-regulated mechanisms by reducing the phosphorylation of downstream mTOR effectors, including 4EBP1 and S6K1, which are responsible for the translation of mRNA encoding pivotal proteins for cell cycle regulation, cell size control, cellular growth, angiogenesis, and glycolytic activity [34].

Despite sharing a central macrolide chemical structure, sirolimus and its analogues yet differ in the functional groups added at C40. While everolimus and ridaforolimus are biochemically active derivatives (hydroxyethyl ester and dimethylphosphinate, resp.) of sirolimus, temsirolimus is a prodrug that is transformed in sirolimus when converted in its active form due to removal of the dihydroxymethyl propionic acid ester group at C40 (Figure 2) [35].

Though sirolimus has been successfully used as an immunosuppressant agent to prevent graft rejection in transplanted patients, the subsequent analogues have been also approved for this indication, as well as to treat other conditions, including in oncology field. Everolimus has been used in posttransplant immunotherapy and in the treatment of breast and renal cancer, as well as in neuroendocrine tumors [36, 37]. Temsirolimus is approved for advanced renal cell carcinoma treatment [38] and ridaforolimus is in advanced stages of clinical development, but it is not yet approved for any specific indication [39].  Table 1 summarizes mentioned drugs' clinical pharmacology and their current medical applications.

### 3.2. Pharmacokinetic Features

Despite the minor differences at C40 between sirolimus and analogues, they have important clinical implications, namely, due to distinct pharmacokinetic features, particularly bioavailability and half-life, as recently reviewed [35, 40].

Sirolimus is orally available as a solution or in tablets and has a high percentage of protein binding (about 92%) and a low oral bioavailability (of about 15%: 14% for solution and 18% for tablets), being rapidly absorbed with an estimated *t*_max_ (time after administration when the maximum plasma concentration is reached) of around 2 hours [35, 41]. The extensive interpatient variability is mainly attributed to the effects of intestinal cytochrome p450 3A enzymes (CYP3A) and P-glycoprotein activity on sirolimus absorption, which are also responsible for some of the drug-drug interactions, the most relevant one being cyclosporine coadministration in renal transplant patients, which increases *C*_max_ (the peak plasma concentration after administration) and area under the curve (AUC) of sirolimus. Sirolimus has a large volume of distribution (around 12 L/kg), being approximately 95% into red blood cells (RBCs). Hepatic CYP3A enzymes are the major metabolizers of sirolimus and elimination occurs predominantly by fecal route (around 90%), with a clearance between 1.45 and 6.93 mL/min/kg and a terminal half-life ranging from 46 to 78 hours [35, 41]. The relative hydrophobicity of sirolimus allows absorption through the skin, which could be an advantage for use in custom topical preparations to treat TSC-related facial angiofibromas [35, 41].

Everolimus is also orally used, once a day, in tablet form. This sirolimus derivative shares some pharmacokinetic features with the original molecule, including the wide distribution into RBCs, the metabolism by hepatic CYP3A enzymes, and the predominantly fecal elimination [35, 41]. However, everolimus is more readily absorbed, exhibits greater oral bioavailability (20%), and has a lower protein binding capacity (around 75%) and several other favorable pharmacokinetic parameters, including a better blood-brain partition coefficient, a greater water solubility, and a shorter half-life, which suggests that a steady-state concentration is achieved more rapidly. Everolimus is rapidly absorbed in healthy volunteers and in patients with solid tumors, with *t*_max_ values of 30 min to 1 hour and half-life of around 30 hours [35, 41]. Thus, everolimus has faster steady-state levels after initiation and faster elimination after withdrawal, which is an advantage over sirolimus.

Temsirolimus is formulated for weekly intravenous administration and was designed to overcome the poor solubility of the prototype mTOR inhibitor, oral rapamycin, which undergoes extensive first-pass metabolism leading to low and potentially variable absorption and exposure. Maximizing the bioavailability and dose intensity by using intravenous administration may provide optimal clinical benefit in some tumors [42]. Temsirolimus exhibits better solubility and an elevated volume of distribution that allows extensive delivery into peripheral tissues. This drug is metabolized by CYP3A4 and is primarily excreted by the feces (around 82%), having a terminal half-live between 9 and 27 hours [35].

Ridaforolimus is a more recent analogue of sirolimus that is being formulated for oral or intravenous administration. Although some pharmacokinetic features are identical to those of sirolimus, including primary metabolization by CYP3A enzymes, preferential elimination by fecal route (around 90%) and terminal half-life ranging from 30 to 75 hours, ridaforolimus shows improved solubility, stability, and bioavailability when compared with sirolimus [43].

## 4. Clinical Trials with mTOR Inhibitors in TSC Patients

### 4.1. Sirolimus

The first clinical study in which sirolimus was used as a TSC treatment was conducted in 2006, when 5 patients with SEGA tumors were submitted to treatment with that mTOR inhibitor for 2.5 to 20 months [44]. Doses were titrated until serum levels of 5–15 ng/mL were reached and an average of 55% reduction in tumor volume was observed [44].

After that, the efficacy and tolerability of sirolimus in TSC patients diagnosed with renal angiomyolipomas and LAM were also investigated in two open-label studies. Twenty-five patients with TSC and LAM were recruited for a proof-of-concept phase I/II study, receiving an initial dose of sirolimus of 0.25 mg/m^2^, followed by periodic uptitrations, until a plasma level of 10–15 ng/mL of the drug was reached [45]. This study showed that renal angiomyolipomas decreased their volumes to approximately 53% of the baseline value after 12 months of treatment, but at 24 months regrowth to 86% of the baseline value was noticed [45].

This set the basis for a Phase II Trial of Efficacy and Safety of Sirolimus for Treatment of Angiomyolipoma in Tuberous Sclerosis and Sporadic LAM (the TESSTAL trial), which involved 16 patients [46, 47]. They initially received the drug with an oral dose of 0.5 mg/m^2^, but it was titrated until a level of 6–10 ng/mL was reached. After 12 months of treatment, a reduction in tumor volume of more than 50% was reported in 80% of the perprotocol group, but it increased again after treatment cessation [46]. Later, at 24 months, a partial response was noticed in 40% of those individuals remaining in the trial [46]. The efficacy of sirolimus in reducing the volume of different types of tumors in patients with LAM was assessed in other studies [47–52]. Additionally, the drug showed also a benefit in the treatment of pulmonary fibrosis and skin manifestations of TSC [53, 54]. Since sirolimus is available in a topical formulation, there is a prospective study in which it was possible to show a reduction in facial angiofibromas in 73% of patients (*n* = 28) [55]. To confirm this, a phase II clinical trial using different doses of topical sirolimus was recently completed (NCT01526356), but the results are not yet available.

In terms of safety, the studies of sirolimus in patients with TSC, namely, with renal angiomyolipomas and LAM showed important rates of adverse events [44–47]. In the proof-of-concept study, infections, diarrhea and aphthous ulcers were the most frequently reported effects [45]. In the phase II trial (TESSTAL study), the majority of adverse events were classified as mild and were consistent with what was already known, from the previous study: mucositis, respiratory infections, and proteinuria were reported [46, 47].

### 4.2. Everolimus

To date, only sirolimus and everolimus have been clinically tested for the management of TSC patients and, in fact, only everolimus is effectively approved for that indication. Following the first report of sirolimus to treat SEGA, two major clinical trials were conducted to test the efficacy and safety of everolimus in that TSC-related condition [56, 57].

The first one was an open-label and prospective study, which recruited 28 patients diagnosed with SEGA and with lesion growth demonstrated on brain magnetic resonance imaging (MRI), performed before study treatment initiation [56]. The median dose of everolimus that was used was 5.3 mg/m^2^/day and the median treatment duration was 34.2 months (range 4.7–47.1). All 28 patients demonstrated a reduction in the volume of the tumors or a cessation of lesion growth. As a measure of efficacy, at 18, 24, 30, and 36 months, primary SEGA volume was reduced ≥30% from baseline (this was the definition of treatment response) in 75% of the patients and more than 30% of them experienced SEGA volume reduction of more than a half within 6 months of treatment [56]. The second study, known as the EXIST-1 trial (Efficacy and Safety of Everolimus [RAD001] in Patients of All Ages With Subependymal Giant Cell Astrocytoma Associated With Tuberous Sclerosis Complex), was a randomized, placebo-controlled, and double-blind study recruiting 117 patients (78 receiving everolimus with a target concentration of 5–15 ng/mL and 39 placebo) and the results were similar to those previously reported: a reduction of more than 50% in SEGA volume was obtained in 49% of patients treated a median of 29 months [57, 58]. Regarding safety issues, in the first study, all patients reported at least one adverse event, but none of them led to everolimus discontinuation: they were grade 1-2 in severity and those most frequently reported were upper respiratory infections, stomatitis, sinusitis, and otitis media [56]. In the EXIST-1 trial, the same situation was observed: patients treated with everolimus had more adverse events than those in the placebo arm, including mouth ulceration, stomatitis, convulsions and pyrexia; three patients aged ≥13 years in the everolimus group experienced amenorrhoea [57].

These studies have also drawn attention to some additional benefits of everolimus (measured as secondary endpoints) for the treatment of other TSC manifestations related to the central nervous system, namely, seizure control and behaviour and cognitive development [56, 59, 60].

The first clinical trial with prospective design to evaluate everolimus efficacy in the treatment of medically refractory epilepsy, in the context of TSC, was published in 2013 and included 23 patients aged ≥2 years [61]. The median dose of everolimus used was 7.5 mg/day and treatment duration was 12 weeks. In terms of efficacy measures, seizure frequency was reduced by 50% or more in 12 of 20 patients and, overall, a median of 73% in reduction of seizures from baseline was reported in 17 out of 20 patients [61]. Several cases experienced an important improvement, considering they had a prior history of failed drugs, vagus nerve stimulation or, inclusively, epilepsy surgery [61]. No new adverse events were reported in this study, but it is worth noting that 3 patients experienced an increase in seizures, highlighting how treatment response may be variable, between different individuals. Complicating this scenario are the results coming from the EXIST-1 trial, in which no significant difference between everolimus and placebo was observed in median seizure frequency at baseline and week 24 [57]. To clarify this situation, two trials are currently under way, aiming to specifically evaluate everolimus' effect on measures of seizure control (NCT01713946) and cognition (NCT01289912: this study is completed, but no results were yet provided) [62].

Everolimus has also been tested in TSC-associated angiomyolipomas and LAM, in the so-called EXIST-2 trial (Everolimus for Angiomyolipoma Associated With Tuberous Sclerosis Complex or Sporadic Lymphangioleiomyomatosis [EXIST-2]: A Multicentre, Randomised, Double-Blind, Placebo-Controlled Trial) [63]. In this study, 118 patients aged 18 years or older were recruited to receive everolimus (*n* = 79) or placebo (*n* = 39). The angiomyolipomas response (defined as at least a 50% reduction in total volume relative to baseline) rate was 42% for those patients treated with everolimus (versus 0% with placebo) and the adverse events were predictable and generally manageable, considering data coming from previous studies [63]. The extension of EXIST-2 was recently published and confirmed a proportion of patients with reductions of ≥30% and ≥50% in angiomyolipoma volume of 82% (62/76) and 65% (49/76), respectively, at week 96, with lesser adverse events over time [64].

## 5. Current Therapeutic Challenges Using mTOR Inhibitors

Sirolimus and everolimus were firstly developed for the treatment of fungal infections and cancer and to prevent organ transplant rejection. This gives a notion about their pharmacological potential, when systemically administered and this may be a theoretical issue, when considering TSC-related features treatable with these agents, since they are, at the end, focal lesion areas. However, this is not straightforward and deserves some considerations.

First of all, robust knowledge about dosing and side effects existed before these drugs were considered to be used in the treatment of TSC. The most frequent adverse events associated with them include stomatitis and mouth ulcers, marrow suppression, infections, hypercholesterolemia, and other metabolic disturbances [65, 66]. TSC patients reported these toxicities in clinical trials, but with overall reduced frequency and severity, comparing with what was theoretically expected [57, 58, 63, 64]. The reason for this can be related to the fact that these agents are used in monotherapy for TSC patients, whereas in other oncological situations and in transplant fields they are combined with other immunosuppressants or chemotherapy. Additionally, in TSC the dosing strategy aims to identify the minimum effective dose, while in other clinical settings (particularly in oncology) dosing is closer to the maximum tolerated, not specifically avoiding side effects [35].

The risk of infection is also an important issue to be discussed, since these drugs have an intrinsic immunosuppressive potential, which is indeed the reason for their use in transplant medicine [67]. Looking at data coming from the first clinical trials in TSC patients, infection rate was reported to be high as 80–90% of treated individuals. Nevertheless, it is worth noting that all infections were recorded as being related to the study drug and the period of patients' follow-up was a minimum of 1 year [56]. In fact, those infection rates actually decreased as patients received treatment for longer and the larger placebo-controlled studies using everolimus in patients with SEGA and with angiomyolipomas came to provide a clearer notion about infection risk: in these studies, only 10–20% of subjects were diagnosed with an infection and this percentage was nearly the same in both the everolimus and placebo arms [57, 63]. Most of those infections were classified as mild or moderate in severity and they were rarely the cause of treatment discontinuation [56, 57, 63, 68].

Finally, but no less relevant, the basis for the choice between sirolimus and everolimus, in clinical practice, should be discussed. There is abundant supportive evidence for the efficacy of both drugs in TSC patients, namely, for the therapeutic approach of some of its manifestations, but the fact is that there are not, to date, clinical trials directly comparing those mTOR inhibitors (in TSC, oncology and transplantation). In the absence of such a study, in TSC patients, clinicians have to follow the best evidence published so far, regarding the selection of a specific drug. In a very recent systematic review, authors concluded that oral everolimus has high-quality evidence regarding its effect in reducing the size of SEGA and adult renal angiomyolipomas, without significantly increasing the risk of patients to experience adverse events, as compared to those not receiving any treatment [69]. However, the usage of the drug seems to increase the risk of dose reduction, interruption, or withdrawal, over time [69]. The existence of a sirolimus topical formulation makes it attractive for the treatment of skin manifestations of TSC, more than the systemic everolimus intake. Nevertheless, topical sirolimus only showed a nonsignificant tendency of skin lesions improvement, meaning that this putative benefit needs to be clarified and further established, as well as the possibility of using these drugs in other TSC clinical features [69].

## 6. Future Directions

Over the past decade, the definition of the multiple roles of the mTOR signalling pathway in neurological conditions has been a successful and exciting story of translational research going from bench to bedside. Beyond gene mutation discovery, several functional experiments were conducted in order to validate the pathological role of those mutations and different pharmacological approaches were developed, trying to manipulate that signalling pathway and allowing benefit to be tested in clinical trials. We have reached a point where some drugs appear very promising in the treatment of some diseases whose manifestations are typically attributed to mTOR pathway dysfunction, but there are a growing number of novel mutations in genes related to components of the mTOR pathway that have recently been linked to several developmental brain malformations. In the same way, changes in mTOR-dependent autophagy have been described and linked to neurological conditions whose pathophysiology is deeply characterized by degenerative processes [70].

Nevertheless, despite all these discoveries, there are some straightforward questions that remain unanswered. How does the dysfunction of the cellular processes depending of mTOR lead to distinct neurological phenotypes? In fact, apart from TSC, developmental brain malformations, autism, and intellectual disability, there are also completely different conditions such as traumatic and hypoxic-ischaemic brain injury, and dementia that were already connected to mTOR dysfunction. How does mTOR activation in different cell types (neurons and glial cells) impact clinical phenotypes, from embryonic brain development to senescence? How can the usage of systemic mTOR inhibitors be optimized, considering that the target could be perfectly contained and localized in some brain areas? How early can treatment with these drugs be (and, regarding this aspect, it is pertinent to think on a comparison of mTOR inhibition and classical antiepileptic drugs, such as vigabatrin, in early manifestations of the disease, such as infantile spasms)? How does mTOR dysfunction allow interaction with other brain signalling pathways, namely, involved in neuronal development?

Regarding this last issue, recent interest has been placed in the dialog between the mTOR and the Sonic Hedgehog (Shh) signalling pathways. Shh is involved in the mechanisms regulating neurogenesis, neuronal migration, and synaptic tuning and a critical step for this molecular pathway is the removal of Ptch1 (transmembrane receptor Patched1) inhibition on the signal transducer Smo (G-protein coupled receptor Smoothened) and its localization in the primary cilium. These cilia are membrane extensions with sensory functions that seem to play a critical role in cell fate definition, considering neuronal progenitors. In a very recent communication, di Nardo et al. [71] presented preliminary data indicating that hyperactivation of mTORC1 signalling is associated with reduced ciliation in* TSC2*-knockdown rat hippocampal neurons, in a neuronal-specific* TSC1*-knockout mouse model and in the brain of TSC patients with epilepsy. Furthermore, the same authors revealed that, in* TSC2*-knockdown neurons, altered Shh signalling was associated with defective ciliation [71]. For the first time, this study suggested that TSC could be considered as a ciliopathy and that Shh/ciliary signalling might represent an additional therapeutic target [71].

Recently, a second generation of mTOR inhibitors (known as mTOR kinase inhibitors or TORKinibs) has also been developed. As a major difference from the rapamycin analogues, they present the ability to directly inhibit the kinase by blocking the ATP catalytic site, rather than linking FKBP12, which causes inhibition of both mTORC1 and mTORC2 [72, 73]. Because of this property, they have been shown distinct implications on downstream target inhibition, as well as on mechanisms of protein translation control and regulation loops. In addition, they would be important to differentiate TORC1- from TORC2-mediated intracellular signalling. Yet, in preclinical or early clinical stages of development, this new generation of mTOR inhibitors are potent inhibitors of proliferation and they are expected to have an important therapeutic benefit in oncological indications [72, 73]. If the dual targeting of TORC1/TORC2 will be able to introduce a superior efficacy when compared to that of everolimus, without further significant toxicity, these newer agents might open new windows of opportunity to the treatment of several conditions, including TSC.

## 7. Conclusions

The treatment of TSC using mTOR inhibitors is an important and promising challenge, from a clinical point of view. Patients have a systemic and progressive disease, but the majority of them develop significant morbidity only by middle age. This means that the decision of using a drug such as a mTOR inhibitor in base of isolated individual organ complications will probably not be the most appropriate strategy and it makes sense, in this field, to use a lifetime risk score to decide how, when, and what to treat. For this approach to be successful, more epidemiological and clinical data need to be collected and studies like the Tuberous Sclerosis Registry to Increase Disease Awareness (TOSCA) registry need to be promoted [74].

Managing TSC requires a multidisciplinary approach and the introduction of mTOR inhibition therapy reinforces the need for this very close collaboration between well-prepared health professionals. Questions remain regarding which drug to choose, the correct timing for treatment initiation and discontinuation, the serum level to be achieved, and the management of long-term side effects. Registries such as TOSCA and further studies will help to answer these pertinent questions.

## Figures and Tables

**Table 1:** Pharmacology and clinical applications of rapamycin (sirolimus) and its analogues, everolimus, temsirolimus, and ridaforolimus.

	Sirolimus	Everolimus	Temsirolimus	Ridaforolimus
Commercial names	Rapamune®	Afinitor®, Votubia®, Certican®, Zortress®, Evertor®	CCI-779, Torisel®	AP23573, MK-8669, Deforolimus

Biochemical features				
*Molecular weight*	914.2 g/mol	958.2 g/mol	1030.3 g/mol	990.2 g/mol
*Mechanism of action*	Inhibition of the TSC-mTOR pathway	Inhibition of the TSC-mTOR pathway	Inhibition of the TSC-mTOR pathway	Inhibition of the TSC-mTOR pathway
*Biochemically functional form*	Sirolimus is the active form	Active derivative (hydroxyethyl ester) of sirolimus	Prodrug actived after removal of the dihydroxymethyl propionic acid ester group at C40 position	Active derivative (dimethylphosphinate) of sirolimus

Pharmacokinetic features				
*Route of administration*	Orally, once daily	Orally, once daily	I.V. infusion, once/week	Oral or intravenous infusion
*Protein binding *	~92%	~75%	~85%	~94%
*Bioavailability and distribution*	Low oral bioavailability (~15%): 14% for solution and 18% for tablets Large distribution (around 12 L/kg), ~95% into RBCs	Tablet: 20% Wide distribution into RBCs; good blood-brain partition coefficient	Injection: 100% Elevated distribution that allows extensive delivery into peripheral tissues	Tablet: 16% Improved solubility, stability and bioavailability vs sirolimus
*Metabolization *	Hepatic CYP3A	Hepatic CYP3A	Hepatic CYP3A4	Hepatic CYP3A4
*Terminal half-life *	46–78 h	26–30 h	9–27 h	30–75 h
*Elimination *	Feces (91%), urine (2%)	Feces (>90%), urine (2%)	Feces (82%), urine (5%)	Feces (88%), urine (2%)

Clinical indications				
*In TSC patients and other indications*	Clinical trials in TSC patients; immunosuppression in transplanted patients	SEGA and adult angiomyolipoma associated with TSC; immunosuppression in transplanted patients; advanced kidney cancer and other tumors (neuroendocrine, breast)	Advanced renal cell cancer	Clinical trials for advanced soft tissue and bone sarcomas and hematologic malignancies
